# Genomic landscape of CCUS compared to MDS and its implications on risk prediction

**DOI:** 10.1038/s41375-024-02273-z

**Published:** 2024-05-10

**Authors:** Sandra Huber, Constance Baer, Stephan Hutter, Natalie Wossidlo, Gregor Hoermann, Christian Pohlkamp, Wencke Walter, Manja Meggendorfer, Wolfgang Kern, Torsten Haferlach, Claudia Haferlach

**Affiliations:** MLL Munich Leukemia Laboratory, Max-Lebsche-Platz 31, 81377 Munich, Germany

**Keywords:** Risk factors, Myelodysplastic syndrome

## To the Editor:

The 5th edition of the WHO classification (WHO HAEM5) newly included myeloid precursor lesions introducing clonal cytopenia of undetermined significance (CCUS) as an entity [1]. CCUS is defined as clonal hematopoiesis (CH) in the presence of unexplained, persistent cytopenia requiring detection of either somatic mutations in certain genes or clonal chromosomal abnormalities. Discriminating CCUS from MDS primarily relies on morphologic bone marrow (BM) dysplasia. However, morphologic criteria are affected by their subjective evaluation and restricted in some cases due to sample quality [2]. The current Revised International Prognostic Scoring System (IPSS-R) for MDS is based on clinical variables and cytogenetic aberrations [3], while more recently cytogenetic and molecular genetic data are integrated with clinical parameters into the Molecular International Prognostic Scoring System (IPSS-M) [4]. The progression risk from CCUS to a myeloid neoplasm (MN) is highly variable [2, 5–7] and depends on various factors such as clone size [8] or number and type of mutations [5, 9]. Thus, models predicting the progression risk in patients with CH were developed, which include besides gene mutations also blood values and various additional parameters (clonal hematopoiesis risk score/CHRS; MN-predict) [10, 11]. However, the strict discrimination of different prognostic systems neglects the continuous development from myeloid precursor lesions to overt MN. Hence, comprehensive genomic studies on the continuum of CCUS and MDS patients are needed to elucidate their underlying molecular heterogeneity and genetic spectrum.

Thus, we analyzed 222 CCUS cases (age: 76 years [26–93]; female: 33%) and 698 MDS cases (age: 73 years [23–93]; female: 43%; Supplementary Table S1). Diagnoses were made following WHO HAEM5 based on cytomorphology, cytogenetics and molecular genetics; in addition WGS and WTS analyses were performed for all patients (for details see Supplement). The IPSS-R, IPSS-M and CHRS were calculated as previously published [3, 4, 10]. Details on statistics are provided in the Supplementary Methods. All patients had given written informed consent to the use of genetic and clinical data, the study was conducted according to the Declaration of Helsinki and approved by the internal review board.

CCUS patients were older, had higher WBC counts, lower PLT counts, higher HB, less BM blasts and were more predominantly male compared to MDS patients (all *p* < 0.05; Supplementary Table S1; Supplementary Figs. S1/S2). Isolated cytopenia was significantly more and pancytopenia less frequent in CCUS than in MDS. The observed differences between CCUS and MDS in terms of the number and types of cytopenias are consistent with other published cohorts [2, 5, 12], which indicate a correlation between BM abnormalities and the severity of cytopenia.

**Fig. 1:**
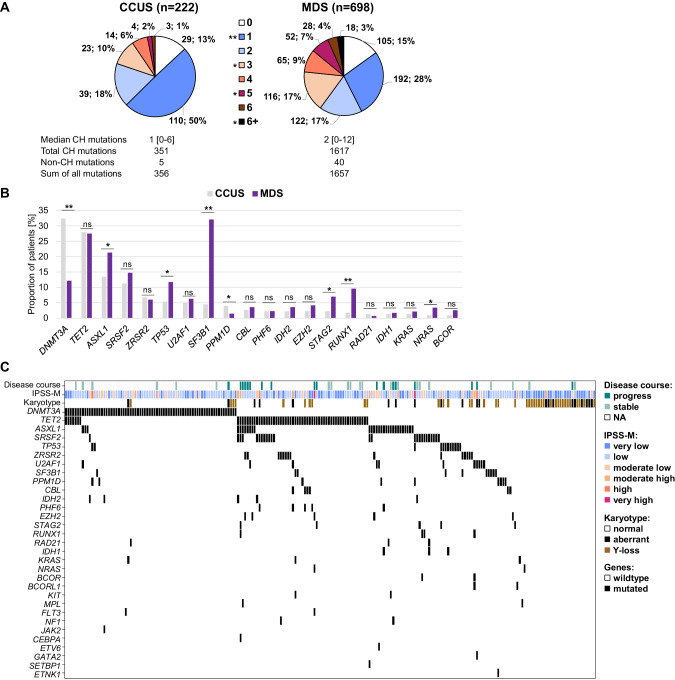
Mutational analysis of CCUS and MDS patients. **A** Distribution of number of CH mutations within CCUS and MDS. CH: clonal hematopoiesis. **B** Frequency of mutated genes in CCUS (gray) or MDS (purple) patients depicting the 20 most frequently mutated genes in CCUS. ** *p* < 0.001; * *p* < 0.05; ns: not significant. **C** Molecular characterization of the CCUS cohort. Illustration of all 222 patients, each column represents one patient. Genes, IPSS-M category, karyotype and follow-up information are given for each patient. NA not available.

**Fig. 2:**
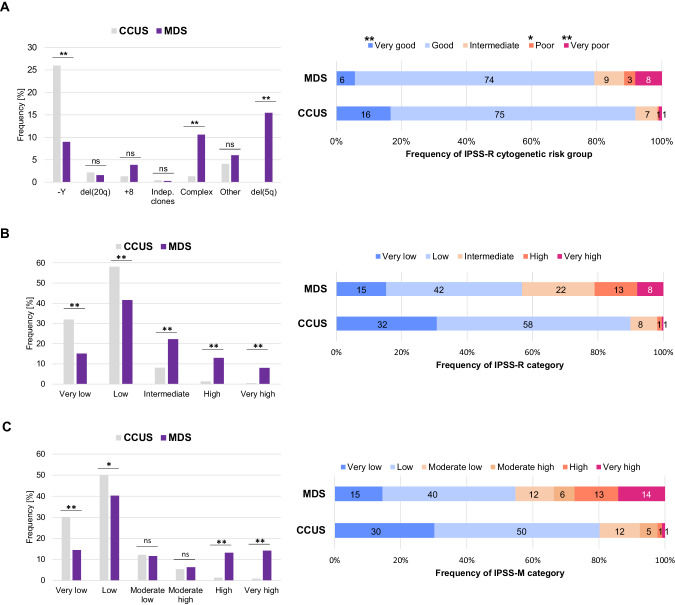
Cytogenetic analysis and risk stratification of CCUS and MDS patients. **A** Frequency of specific chromosomal abnormalities in CCUS and MDS patients (left panel) and distribution of the five IPSS-R cytogenetic risk groups within CCUS and MDS patients. Frequency of patients with Y-loss was adjusted for males only. Indep.: independent. **B** Distribution of the five IPSS-R risk categories within CCUS and MDS patients. **C** Distribution of the six IPSS-M risk categories within CCUS and MDS patients. ** *p* < 0.001; * *p* < 0.05; ns not significant.

Three hundred and fifty-six somatic mutations were found within the entire CCUS cohort, with 351 mutations detected in 28 CH genes (Fig. 1A; Supplementary Table S2; Supplementary Fig. S3/S4). Overall, CCUS patients had fewer CH mutations per patient compared to MDS (*p* < 0.001; Fig. 1A) comparable to a previous study [2]. It was further shown that in MDS, the number of mutations increases with the number of BM blasts and the severity of the disease [4]. CCUS patients most frequently harbored mutations in *DNMT3A* (32%), *TET2* (28%) and *ASXL1* (14%) followed by mutations in splicing factor genes and *TP53* (Fig. 1B/C; Supplementary Fig. S4). Overall, mutations in *DNMT3A* and *PPM1D* were more frequent in CCUS than in MDS while mutations in *ASXL1, TP53, SF3B1, STAG2, RUNX1, NRAS, CUX1* were less frequent in CCUS (each *p* < 0.05; Fig. 1B; Supplementary Table S2). The respective variant allele frequencies (VAF) and the association with blood count alterations are described in the Supplement. In addition, multi-hit *TP53* cases (*n* = 2; *TP53* VAF: 5% + 3%; 25% + 18%) were less frequent in CCUS compared to MDS (*p* = 0.027; Supplementary Table S3). Of note, neither deletions nor CN-LOH involving *TP53* were detected in multi-hit *TP53* CCUS in contrast to MDS. However, 9 recurrent CN-LOH (4q: *n* = 4, of which 3 included *TET2*; 7q: *n* = 5, of which 2 included *EZH2*) were observed in the CCUS cohort. In addition to the influence of the number and type of mutations on the risk of survival and progression in CCUS and MDS patients [2, 4, 5, 10, 11], Gao et al. demonstrated that chromosomal alterations influence disease progression in CHIP/CCUS independently of the other parameters [9]. Cytogenetic abnormalities were detected in 27% of CCUS and 43% of MDS patients (*p* < 0.001) with loss of chromosome Y (Y-loss) being the most common (Supplementary Results; Supplementary Fig. S7). Y-loss was more frequent in CCUS than in MDS (males: 26% vs. 9%; *p* < 0.001), while complex karyotypes and del(5q) were more frequent in MDS (both *p* < 0.001; Fig. 2A; Supplementary Fig. S7). This is consistent with the fact that Y-loss is described as the most common acquired cytogenetic alteration in aging males that is not associated with progression to MN [13]. MDS patients frequently had both mutations and chromosomal aberrations (37%), while only 14% of CCUS patients had both types of aberrations (*p* < 0.001; Suppl. Results; Supplementary Fig. S9). The co-occurrence of chromosomal alterations and mutations were also reported to be rare in myeloid CH cases [9, 14] while the combination of both types of aberrations is substantially more frequent in overt MN.

Applying the IPSS-R cytogenetic risk groups, 92% of CCUS patients were assigned into the very good or good cytogenetic risk group (MDS: 79%; Fig. 2A; Supplementary Table S1/Fig. S11). The final IPSS-R categories showed significant differences in all categories between CCUS and MDS (Supplementary Table S1; Fig. 2B). Also the IPSS-M categories showed a clear skewing towards low risk categories in CCUS (Fig. 2C; Supplementary Fig. S12). In the entire CCUS cohort, only 5 cases (2%) were assigned to IPSS-M high or very high risk groups harboring a median number of 4 mutations and including both multi-hit *TP53* patients. This skewing towards lower risk categories is plausible, was also recently described in another CCUS cohort [15], and partly reflects the fact that patients with increased blasts are by definition excluded from the CCUS category. Nevertheless, some CCUS patients were categorized as IPSS-M high risk, suggesting that the IPSS-M may also be applicable in CCUS.

Finally, in an exploratory analysis, we asked whether the risk categories lead to a meaningful survival prediction in CCUS patients (Supplementary Fig. S13/ Table S7). During the relatively short period of observation, IPSS-R risk stratification did not show a significant overall survival (OS) difference between very low/low and the remaining cases (Supplementary Fig. S13B), while IPSS-M showed a trend to differentiate OS between very low/low and the remaining CCUS patients (*p* = 0.059; Supplementary Fig. S13C, D) roughly comparable to that of MDS patients in the respective risk groups. This is consistent with a previous study [6] that demonstrated clinical and molecular overlap between CCUS and low risk MDS patients. In summary, despite the relatively short 1.5 years median follow-up and the underrepresentation of moderate and high risk cases in the CCUS cohort, survival data of CCUS patients stratified according to the IPSS-M seem meaningful. For a subset of cases, we also calculated the CHRS. In contrast to IPSS-R and IPSS-M, distribution of CHRS categories was more evenly (Supplementary Table S7). No significant differences in OS of the CCUS cohort stratified for CHRS groups were observed which might be due to missing values and thus the inability for precise grouping and due to small sample sizes (Supplementary Fig. S13E, F). For a small subset of patients, multiple samples were taken during disease course (Suppl. Results). In line with other studies [10, 11], we also observed that a proportion of CCUS patients progressed to an overt MN. Interestingly, progressing patients more frequently harbored *ASXL1* mutations than non-progressing patients (*p* = 0.035). However, *ASXL1* mutations are not regarded in the CHRS, also not considering cytogenetic abnormalities and types of cytopenias.

The current risk scores (IPSS-R [3], IPSS-M [4], CHRS [10], MN-predict [11]) were developed either to determine the risk of progression from CH to MN or from MDS to AML and thus do not take the biological continuum into account. So far, the distinction between CCUS and MDS must first be made on the basis of the rather subjective assessment of BM dysplasia, and then the corresponding risk score can be applied. Limitations of our study include the retrospective design, the low number of patients with complete clinical data and the relatively short median follow-up of the CCUS cohort. Thus, additional prospective studies on CCUS patients are needed to validate our observations. In summary, our data confirm the comparable mutational spectrum between CCUS and MDS reflecting the biological continuum but clearly show major differences in the frequency and VAF of distinct gene mutations. These biological differences hint towards different subgroups within CCUS with cases closer to MDS than others. A combined and easy-applicable risk score for MDS and CCUS would reflect this continuous spectrum and has the potential to derive an objective risk assessment irrespective of observer-dependent grading of dysplasia.

### Supplementary information

Supplement

## Data Availability

The datasets generated during and/or analyzed during the current study are available from the corresponding author on reasonable request.
