# Supplementary material for: Genomic landscape of CCUS compared to MDS and its implications on risk prediction
Source: Leukemia. 2024 May 10;38(7):1634–7. doi: 10.1038/s41375-024-02273-z (PMC11216976; doi:10.1038/s41375-024-02273-z)
Supplement: Supplementary file 1 — Supplement [file 41375_2024_2273_MOESM1_ESM.docx]

**Supplementary Material**

***Patients cohort and samples***

All CCUS samples were sent to the MLL Munich Leukemia Laboratory between 03/2019 and 12/2021, MDS samples (included in [1]) between 09/2005 and 01/2020. Diagnoses (from peripheral blood (PB) and BM) were made based on cytomorphology, cytogenetics and molecular genetics as published [2-4]. Genetic data were not available to the cytomorphologist. Therapy-related cases were excluded. Anemia was defined as hemoglobin/HB <13 g/dL in males and <12 g/dL in females, thrombocytopenia as platelets/PLT <150x10^9^/L, and neutropenia as absolute neutrophil count/ANC <1.8x10^9^/L. To exclude other causes of cytopenia, additional immunophenotyping [5] was carried out if neccessary. Clinical follow-up data was available in 73/222 CCUS and 693/698 MDS patients. A follow-up sample was available for 39/222 CCUS patients.

**Supplementary Methods**

***Whole genome and whole transcriptome sequencing (WGS, WTS)***

For WGS (median coverage 100x), total genomic DNA was extracted from lysed cell pellet of BM or PB using the MagNA Pure 96 with DNA and Viral Nucleic Acid Large Volume Kit and Cellular RNA Large Volume Kit (Roche, Basel, Switzerland). Library preparation and sequencing as well as calling and filtering of single nucleotide variants, structural variants and somatic copy number variations (CNVs) were performed as previously described [6, 7]. Copy neutral loss of heterozygosity (CN-LOH) was assessed using HadoopCNV [8]. WTS (50 Mio reads) was performed as described therein [9]. Fusion calling was performed with Manta (v0.29.0) [10], Arriba (v1.2.0) [11] and STAR-Fusion (v1.9.0) [12].

#### Mutational analysis

Mutational data was retrieved during routine work-up using targeted panel next generation sequencing [4] (median coverage 1500x; performed for all patients) or from WGS data. The mutation status of 59 genes associated with myeloid malignancies (54 CH associated genes based on WHO HAEM5 + 5 additional IPSS-M genes) were analyzed. In detail, we evaluated 54 genes associated with clonal hematopoiesis (CH genes: *ASXL1, BCOR, BCORL1, CBL, DNMT3A, IDH1, IDH2, JAK2, KRAS, NRAS, PPM1D, SF3B1, SRSF2, TET2, TP53, U2AF1, CEBPA, ETV6, EZH2, GATA2, KIT, MPL, PHF6, RAD21, RUNX1, SETBP1, STAG2, ZRSR2, BRCC3, CTCF, GNAS, GNB1, PTPN11, BRAF, CALR, CREBBP, CSF3R, CUX1, KDM6A, NOTCH1, PIGA, SF1, SF3A1, SMC1A, SMC3, U2AF2, WT1, CSF1R, JAK3, KMT2A, MYD88, PRPF40B, PTEN, STAT3*) and 5 genes required in addition for calculating the IPSS-M (non-CH genes: *ETNK1, FLT3, NF1, PRPF8, NPM1*). A VAF cut-off of 2% (or 4% for X-chromosomal genes in men) was used as defined in the WHO classification. The presence of *FLT3*-ITD and *KMT2A*-PTD were retrieved from WGS data only. Structural variants/ fusions were analyzed by combining routine cytogenetics (encompassing chromosome banding analyses/ CBA and FISH) and WGS/WTS.

#### CHRS calculation

The CHRS was calculated for CCUS cases with available follow-up data (n=73). For 8 cases only showing cytogenetic abnormalities without somatic mutations the CHRS was not calculable. Due to the retrospective design of the study parameters MCV (mean corpuscular volume) and RDW (red cell distribution width) were not always available. Therefore, in 47/73 CCUS patients the CHRS could not be calculated. Nevertheless in 17 cases, missing values did not impact risk categorization, as even with the lowest possible or highest possible scores for missing values, the risk category of these cases would not have changed. However, in 30 cases even the risk category could not be determined precisely, as the lowest possible or highest possible scores for missing values led to categorization in different risk groups. Patients at the border between low and intermediate risk that might qualify for the intermediate risk group if missing values were available were termed “intermediate^pot^”. Patients at the border between intermediate and high risk that might qualify for the high risk group if missing values were available were termed “high^pot^”.

***Statistical analysis***

For statistical analyses SPSS version 19.0 (IBM Corporation, Armonk, NY) was used. Analyses for overall survival (OS) were performed according to Kaplan-Meier and compared using two-sided log rank tests. The OS was calculated as time from diagnosis to death or last follow-up. Dichotomous variables were compared using Fishers exact or chi-square test. For comparison of median values, the two-sided Mann-Whitney U test was applied. All results were considered significant at *p*<0.05. *P*-values were adjusted for multiple testing following Benjamini-Hochberg [13] where appropriate. For density plots R version 4.3.0 (R Foundation for Statistical Computing, Vienna, Austria) was used.

**Supplementary Results**

***Mutation detection by NGS panel sequencing compared to WGS***

Of all 356 MUT found by NGS panel sequencing, 10 (3%) were not detected by WGS (median NGS VAF: 3% [2-5%]), whereas 3 MUT were only seen by WGS (median WGS VAF: 19% [6-50%]) as corresponding exons were not covered by NGS panel sequencing (Suppl. Figure S3). Considering WGS data only, 6 patients would not have been classified as CCUS as their sole mutation was not found by WGS.

***Analysis of variant allele frequencies/VAF***

Within the 15 most frequently mutated genes in CCUS (found in ≥4 patients), mutations in 6 genes (*DNMT3A, SF3B1, TP53, CBL, STAG2, PPM1D)* showed a median VAF <10% (Suppl. Figure S5). Ten of the top 15 genes showed significantly lower median VAF than in MDS (*DNMT3A, TET2, ASXL1, SRSF2, U2AF1, SF3B1, TP53, PPM1D, IDH2, STAG2;* each *p*<0.05; Suppl. Figure S6; Suppl. Table S4). Thus, the frequency and VAF of *ASXL1*, *TP53*, *SF3B1 and STAG2* mutations were significantly lower in CCUS than in MDS, while *DNMT3A* and *PPM1D* mutations were detected significantly more often in CCUS, but at a lower VAF than in MDS.

***Detailed analysis of cases with Y-loss***

Focusing on cases with Y-loss (CCUS: n=39; MDS: n=37), the median proportion of aberrant metaphases/interphase nuclei was lower in CCUS cases than in MDS cases independent of the cytogenetic detection method (chromosome banding analysis/CBA: 50% vs. 85%, *p*<0.001; FISH: 45% vs. 75%, *p*<0.001; Suppl. Figure S8). For both entities chromosome banding analysis (CBA) revealed slightly higher proportion of aberrant cells than FISH (CCUS: 50% vs. 45%, *p*=0.036; MDS: 85% vs. 79%, *p*=0.033; Suppl. Figure S8).

Of note, based on WGS data Y-loss was found in additional 7 patients (median proportion of aberrant interphase nuclei by FISH: 34% [11-52%]), for which CBA revealed normal karyotypes in the presence of somatic mutations, while in 9 patients chromosomal abnormalities retrieved from CBA were not detected by WGS (affecting 3 patients with chromosomal abnormalities only and 6 with additional somatic mutations; Suppl. Table S5).

***Analysis of genetic clonality***

Analyzing the type of genetic clonality (somatic mutation and/or chromosomal abnormality) within the CCUS cohort, 29 patients (13%) showed cytogenetic abnormalities only (thereof 69% Y-loss), while the remaining 193 patients (87%) showed at least one mutation in any of the 54 CH associated genes. Of these, 162 (84% of 193; 73% of all) harbored mutations only, while 31 (16% of 193; 14% of all) showed mutations and cytogenetic abnormalities (Suppl. Figure S9). Of note in MDS, the distribution of all three groups was significantly different. Patients with only CH mutations and patients with only chromosomal abnormalities were less frequent in MDS than in CCUS (48% vs. 73%: *p*<0.001 and 6% vs. 13%: *p*=0.002, respectively). In MDS patients with exclusively chromosomal abnormalities, the most frequent one was del(5q) (52%), not detected in any CCUS patient. Patients harboring CH mutations and chromosomal abnormalities in combination were more frequent in MDS compared to CCUS (37% vs. 14%: *p*<0.001). Interestingly, 9% of MDS patients (n=61; 42 MDS-LB, 19 MDS-IB) showed neither CH mutation nor chromosomal abnormalities (thereof 4 had somatic mutations in other myeloid genes: *DDX41* (n=2), *DDX54, FANCL)*.

***Association of genotype and phenotype***

With regard to associations between cytopenias and somatic mutations in CCUS patients, the presence of neutropenia was significantly associated with *TET2* mutations (adjusted *p*<0.001; Suppl. Table S6). In MDS, several significant associations were detected (Suppl. Table S6). Isolated anemia was significantly associated with mutated *SF3B1* but wildtype *TET2, SRSF2, TP53, U2AF1, STAG2, RUNX1* (all adjusted *p*<0.05). In contrast, pancytopenia was significantly associated with *TP53, STAG2* and *BCOR* mutations and wildtype *SF3B1*. In both CCUS and MDS, the number of cytopenias significantly correlated with the number of mutations (Kendall's rank correlation: *p*=0.012 for CCUS; *p*<0.001 for MDS; Suppl. Figure S10).

***Transcriptomic analysis***

In the entire CCUS cohort, no gene fusion transcripts were detected by WTS while in the MDS cohort, in two patients gene fusion transcripts including a gene associated with myeloid malignancies were found (*RUNX1*::*VWF*; *RUNX1*::*ZFPM2*).

***Detailed IPSS-M analysis***

CCUS patients of VL and L risk groups (80%; n=178) showed a median number of 1 mutation and also included 26 patients exclusively harboring chromosomal abnormalities. Of note, 81% (58/72) of patients with mutated *DNMT3A* were VL or L, thereof 40 patients solely harboring *DNMT3A* mutations. In addition, VL and L categories also included 95% (37/39) of patients with Y-loss.

***Detailed follow-up analysis***

For 39/222 CCUS patients multiple samples were acquired during disease course (Figure 1C). Progression to an overt myeloid neoplasm (MDS or AML) was observed in 46% (n=18) showing a median progression free survival of 1.4 years (range: 0.4-3.4 years). However, the majority of these cases (72%) were assigned to IPSS-M low-risk (11 VL, 2 ML), while the CHRS of progressing patients mostly (88%) indicated high risks (8 high, 7 high^pot^; Suppl. Table S8). Interestingly, progressing patients harbored in total more mutations (median: 3 vs. 1; *p*=0.014) and more frequently showed *ASXL1* mutations (50% [9/18] vs. 14% [3/21]: *p*=0.035). In detail, progressing patients (n=18) harbored in median 3 mutations (range: 0-6) while those remaining in stable disease (n=21) had in median one mutation (range: 0-5). Interestingly, the majority of progressing patients were assigned to low risk based on IPSS-R and IPSS-M, while regarding the CHRS the majority of progressing patients were at high risk (Suppl. Table S8). Patients remaining in stable disease were mostly assigned as low risk based on IPSS-R or IPSS-M (Suppl. Table S8). Notably, 9/18 progressing patients harbored *ASXL1* mutations in contrast to 3 of 21 of non-progressing patients (*p*=0.035). Even though 11 progressing samples were assigned as low risk based on IPSS-M, 7 of them also harbored mutated *ASXL1* and a median number of 3 mutations. However, molecular data over time was only available in 23/39 patients (examples are shown in Suppl. Figures S14/S15) including 4 of the 5 patients assigned as very high or high risk according to the IPSS-M. Interestingly, 3 patients (2 VH, 1 H) showed disease progression to MDS and/or AML (VH patient A: 1.4 years to MDS; VH patient B: 0.5 years to MDS, 0.8 years to AML; H patient C: 1.4 years to AML; Suppl. Figure S14A-C). The other high risk patient (D) remained in stable disease harboring multi hit *TP53*, however with VAF of both individual *TP53* mutations was below 10% (Suppl. Figure S14D). Interestingly, patient B and D (IPSS-M VH and H) were grouped into IPSS-R low and very low categories, respectively, not including molecular genetics.

**Supplementary Tables and Figures**

**Table S1. Patients’ characteristics**

| Characteristics | CCUS (n=222) | MDS (n=698) | *p*-value |
| --- | --- | --- | --- |
| **Age** (years; median [range]) | 76 [26-93] | 73 [23-93] | 0.004 |
| **Sex** (female/male: n, %) | 74 (33%) / 148 (67%) | 297 (43%) / 401 (57%) | 0.015 |
| **Bone marrow blast count** (%; median [range]) | 2 [0 - 4.5] | 4 [0 - 19] | <0.001 |
| **WBC**  (median with range; in µl) | 5,100 (1,100-13,800) | 4,600 (300-82,900) | 0.031 |
| **HB**  (median with range; g/dl) | 11.1 (5.6-19.9) | 10.0 (4.4-19.6) | <0.001 |
| **PLT**  (median with range; in µl) | 128,500  (3,000 - 602,000) | 172,000  (2,000 - 1,491,000) | <0.001 |
| **Cytopenia** – data availability | n=222 | n=651 |  |
| Anemia | 158 (71%) | 608 (93%) | <0.001 |
| Thrombocytopenia | 132 (60%) | 297 (46%) | <0.001 |
| Neutropenia | 41 (19%) | 230 (35%) | <0.001 |
| Single lineage cytopenia: | 125 (56%) | 287 (44%) | 0.003 |
| Isolated anemia | 80 (36%) | 265 (41%) | 0.280 |
| Isolated  thrombocytopenia | 40 (18%) | 14 (2%) | <0.001 |
| Isolated neutropenia | 5 (2%) | 8 (1%) | 0.363 |
| Bicytopenia: | 85 (38%) | 244 (38%) | 0.873 |
| Thrombocytopenia +  anemia | 61 (28%) | 142 (22%) | 0.130 |
| Thrombocytopenia +  neutropenia | 19 (9%) | 21 (3%) | 0.004 |
| Anemia + neutropenia | 5 (2%) | 81 (12%) | <0.001 |
| Pancytopenia | 12 (5%) | 120 (18%) | <0.001 |
| **Cytogenetics** | n=222 | n=698 |  |
| Aberrant karyotype (n; %) | 60 (27%) | 301 (43%) | <0.001 |
| -Y | 39 (26% of males) | 37 (9% of males) | <0.001 |
| del(20q) | 5 (2%) | 11 (2%) | 0.563 |
| +8 | 3 (1%) | 27 (4%) | 0.130 |
| Independent clones | 1 (0%) | 2 (0%) | 0.563 |
| Complex (≥3  abnormalities) | 3 (1%) | 74 (11%) | <0.001 |
| Other | 9 (4%) | 42 (6%) | 0.419 |
| del(5q) | 0 (0%) | 108 (16%) | <0.001 |
| **IPSS-R cytogenetic risk group** | n=222 | n=698 | <0.001 |
| Very good | 37 (16%) | 40 (6%) |  |
| Good | 167 (75%) | 513 (74%) |  |
| Intermediate | 15 (7%) | 64 (9%) |  |
| Poor | 1 (1%) | 24 (3%) |  |
| Very poor | 2 (1%) | 57 (8%) |  |
| **IPSS-R risk category** | n=222 | n=661 | <0.001 |
| Very low (VL) | 71 (32%) | 100 (15%) |  |
| Low (L) | 129 (58%) | 275 (42%) |  |
| Intermediate (I) | 18 (8%) | 147 (22%) |  |
| High (H) | 3 (1%) | 86 (13%) |  |
| Very high (VH) | 1 (1%) | 53 (8%) |  |
| **IPSS-M risk category** | n=222 | n=698 | <0.001 |
| Very low (VL) | 67 (30%) | 101 (15%) |  |
| Low (L) | 111 (50%) | 281 (40%) |  |
| Moderate low (ML) | 27 (12%) | 81 (12%) |  |
| Moderate high (MH) | 12 (6%) | 44 (6%) |  |
| High (H) | 3 (1%) | 92 (13%) |  |
| Very high (VH) | 2 (1%) | 99 (14%) |  |
| **WHO subgroup** |  |  |  |
| MDS-*SF3B1* | - | 160 (23%) |  |
| MDS-5q | - | 98 (14%) |  |
| MDS-*TP53* | - | 40 (6%) |  |
| MDS-LB | - | 138 (20%) |  |
| MDS-IB1 | - | 144 (20%) |  |
| MDS-IB2 | - | 118 (17%) |  |
| **Clinical data** |  |  |  |
| Treatment data - availability | 58 (26%) | 651 (93%) |  |
| Intensive chemotherapy | 3* (5%) | 44 (7%) |  |
| Not intensive  chemotherapy | 0 (0%) | 182 (28%) |  |
| Supportive treatment | 13 (23%) | 242 (37%) |  |
| None | 42 (72%) | 183 (28%) |  |

WBC: white blood cells; HB: hemoglobin; PLT: platelets; CH: clonal hematopoiesis; LB/IB: low/increased blasts. *after progress to MDS (n=2) or AML (n=1)

**Table S2. Frequency of mutations detected in CCUS compared to MDS**

|  | CCUS pts (n=222) | | | MDS pts (n=698) | | |  |
| --- | --- | --- | --- | --- | --- | --- | --- |
| **Mutated gene** | **MUT (n)** | **Pts (n)** | **Pts (%)** | **MUT (n)** | **Pts (n)** | **Pts (%)** | ***p*-value (pts)** |
| *DNMT3A** | 78 | 72 | 32.4 | 89 | 85 | 12.2 | **<0.001** |
| *TET2* | 94 | 62 | 27.9 | 298 | 192 | 27.5 | 0.931 |
| *ASXL1** | 30 | 30 | 13.5 | 154 | 149 | 21.3 | **0.011** |
| *SRSF2* | 26 | 25 | 11.3 | 103 | 103 | 14.8 | 0.153 |
| *ZRSR2* | 15 | 15 | 6.8 | 45 | 42 | 6.0 | 0.753 |
| *TP53** | 14 | 12 | 5.4 | 100 | 82 | 11.7 | **0.005** |
| *U2AF1* | 13 | 11 | 5.0 | 44 | 44 | 6.3 | 0.519 |
| *SF3B1** | 12 | 10 | 4.5 | 229 | 224 | 32.1 | **<0.001** |
| *PPM1D** | 11 | 9 | 4.1 | 11 | 10 | 1.4 | **0.030** |
| *CBL* | 7 | 6 | 2.7 | 31 | 25 | 3.6 | 0.672 |
| *PHF6* | 6 | 5 | 2.3 | 20 | 16 | 2.3 | 1 |
| *IDH2* | 5 | 5 | 2.3 | 25 | 25 | 3.6 | 0.393 |
| *EZH2* | 5 | 5 | 2.3 | 33 | 29 | 4.2 | 0.224 |
| *STAG2** | 5 | 5 | 2.3 | 60 | 49 | 7.0 | **0.005** |
| *RUNX1** | 4 | 4 | 1.8 | 80 | 67 | 9.6 | **<0.001** |
| *RAD21* | 3 | 3 | 1.4 | 5 | 5 | 0.7 | 0.416 |
| *IDH1* | 3 | 3 | 1.4 | 12 | 12 | 1.7 | 1 |
| *KRAS* | 3 | 3 | 1.4 | 15 | 15 | 2.1 | 0.585 |
| *NRAS** | 3 | 2 | 0.9 | 26 | 24 | 3.4 | **0.039** |
| *BCOR* | 3 | 2 | 0.9 | 20 | 18 | 2.6 | 0.188 |
| *BCORL1* | 2 | 2 | 0.9 | 3 | 3 | 0.4 | 0.602 |
| *KIT* | 2 | 2 | 0.9 | 4 | 4 | 0.6 | 0.64 |
| *MPL* | 2 | 2 | 0.9 | 11 | 11 | 1.6 | 0.746 |
| *FLT3* | 2 | 2 | 0.9 | 9 | 7 | 1.0 | 1 |
| *NF1* | 2 | 2 | 0.9 | 18 | 17 | 2.4 | 0.185 |
| *JAK2* | 1 | 1 | 0.5 | 16 | 16 | 2.3 | 0.088 |
| *CEBPA* | 1 | 1 | 0.5 | 17 | 15 | 2.1 | 0.139 |
| *ETV6* | 1 | 1 | 0.5 | 14 | 14 | 2.0 | 0.134 |
| *GATA2* | 1 | 1 | 0.5 | 9 | 8 | 1.1 | 0.465 |
| *SETBP1* | 1 | 1 | 0.5 | 9 | 8 | 1.1 | 0.465 |
| *ETNK1* | 1 | 1 | 0.5 | 12 | 12 | 1.7 | 0.208 |

MUT: mutation; pts: patients; n: number of samples; significant *p*-values are marked in bold, and the corresponding genes with (*)

**Table S3. *TP53* mutations in CCUS compared to MDS**

|  | CCUS | MDS |
| --- | --- | --- |
| *TP53* mutated patients (n) | 12 | 82 |
| Single mutation (n) | 10 (83%) | 37 (45%) |
| Multi hit (n) | 2 (17%) | 45 (55%) |
| ≥2 MUT (n) | 2 | 16 |
| MUT+del (n) |  | 17 |
| MUT+CN-LOH (n) |  | 12 |

MUT: mutation; del: deletion; CN-LOH: copy neutral loss of heterozygosity; n: number of samples

**Table S4. VAF analysis of top 15 CCUS mutations compared to MDS**

|  | VAF (%) in CCUS | | VAF (%) in MDS | |  |
| --- | --- | --- | --- | --- | --- |
| **Mutated gene** | Median | Average | Median | Average | *p*-value |
| *TET2** | 25 | 26 | 39 | 36 | **<0.001** |
| *DNMT3A** | 6 | 10 | 35 | 28 | **<0.001** |
| *ASXL1** | 14 | 19 | 28 | 27 | **0.003** |
| *SRSF2** | 37 | 30 | 43 | 39 | **0.001** |
| *ZRSR2* | 29 | 27 | 37 | 30 | 0.256 |
| *TP53** | 5 | 8 | 38 | 37 | **<0.001** |
| *U2AF1** | 17 | 20 | 39 | 33 | **0.006** |
| *SF3B1** | 6 | 9 | 38 | 35 | **<0.001** |
| *PPM1D** | 5 | 8 | 25 | 25 | **0.003** |
| *CBL* | 6 | 10 | 16 | 19 | 0.509 |
| *PHF6* | 10 | 16 | 11 | 20 | 0.692 |
| *IDH2** | 29 | 24 | 44 | 37 | **0.048** |
| *EZH2* | 50 | 48 | 29 | 35 | 0.45 |
| *STAG2** | 6 | 10 | 19 | 23 | **0.036** |
| *RUNX1* | 21 | 21 | 31 | 28 | 0.488 |

VAF: variant allele frequency; significant *p*-values are marked in bold, and the corresponding genes with (*); VAF of X-linked genes in males was corrected for gender.

**Table S5. Discrepant cases regarding cytogenetic analysis from CBA vs. WGS**

| Samples | CH mutations | CBA | FISH | Detected by WGS | Missed by WGS |
| --- | --- | --- | --- | --- | --- |
| 1 | yes | normal | -Y: 47% | -Y |  |
| 2 | yes | normal | -Y: 34% | -Y |  |
| 3 | yes | normal | -Y: 30% | -Y |  |
| 4 | yes | normal | -Y: 20% | -Y |  |
| 5 | yes | normal | -Y: 52% | -Y |  |
| 6 | yes | normal | -Y: 35% | -Y |  |
| 7 | yes | normal | -Y: 11% | -Y |  |
| 8 | yes | del(20q) | del(20q): 5% |  | del(20q) |
| 9 | yes | complex >3 | 7% |  | complex |
| 10 | yes | -Y | -Y: 18% |  | -Y |
| 11 | yes | +8 | +8: 4% |  | +8 |
| 12 | no | -Y | -Y: 12% |  | -Y |
| 13 | yes | +8 | +8: 2.5% |  | +8 |
| 14 | yes | t(18;20) |  |  | t(18;20) |
| 15 | no | del(9q) | del(9q): 4% |  | del(9q) |
| 16 | no | -X | -X: 8% |  | -X |

CH: clonal hematopoiesis; CBA: chromosome banding analysis

**Table S6. Significant associations between cytopenias and genes**

| Cohort | Cytopenia | Gene | Adjusted *p* | % mutated in cytopenia | % mutated in non- cytopenia | Direction |
| --- | --- | --- | --- | --- | --- | --- |
| CCUS | Ne | *TET2* | <0.001 | 58.5 | 21.0 | MUT |
| MDS | An | *SF3B1* | 0.001 | 35.2 | 7.0 | MUT |
| MDS | An | *SRSF2* | 0.040 | 13.5 | 32.6 | WT |
| MDS | An | *TET2* | <0.001 | 25.7 | 58.1 | WT |
| MDS | Iso An | *SF3B1* | <0.001 | 56.6 | 17.4 | MUT |
| MDS | Iso An | *SRSF2* | <0.001 | 5.3 | 21.2 | WT |
| MDS | Iso An | *STAG2* | <0.001 | 1.9 | 10.9 | WT |
| MDS | Iso An | *TET2* | 0.020 | 20.8 | 32.6 | WT |
| MDS | Iso An | *TP53* | 0.016 | 6.4 | 15.0 | WT |
| MDS | Iso An | *U2AF1* | 0.035 | 3.0 | 9.1 | WT |
| MDS | Iso An | *RUNX1* | 0.017 | 5.3 | 13.2 | WT |
| MDS | Iso Ne | *ZRSR2* | 0.017 | 50.0 | 5.8 | MUT |
| MDS | Ne | *SF3B1* | <0.001 | 20.0 | 40.6 | WT |
| MDS | Ne | *STAG2* | <0.001 | 13.5 | 3.8 | MUT |
| MDS | Pan | *SF3B1* | <0.001 | 15.0 | 37.5 | WT |
| MDS | Pan | *STAG2* | <0.001 | 18.3 | 4.7 | MUT |
| MDS | Pan | *BCOR* | 0.035 | 7.5 | 1.7 | MUT |
| MDS | Pan | *TP53* | 0.038 | 20.0 | 9.6 | MUT |
| MDS | Th | *SF3B1* | <0.001 | 13.1 | 50.3 | WT |
| MDS | Th | *SRSF2* | <0.001 | 25.9 | 5.4 | MUT |
| MDS | Th | *NRAS* | 0.010 | 6.4 | 1.1 | MUT |
| MDS | Th | *RUNX1* | <0.001 | 15.8 | 5.1 | MUT |
| MDS | Th | *STAG2* | 0.016 | 11.1 | 4.0 | MUT |
| MDS | Th | *DNMT3A* | 0.020 | 7.4 | 15.8 | WT |
| MDS | Th | *TP53* | 0.009 | 16.5 | 7.3 | MUT |
| MDS | Th+An | *RUNX1* | <0.001 | 20.4 | 7.1 | MUT |
| MDS | Th+An | *SF3B1* | <0.001 | 14.1 | 38.7 | WT |
| MDS | Th+An | *SRSF2* | <0.001 | 26.8 | 11.4 | MUT |
| MDS | Th+An | *ASXL1* | 0.035 | 32.4 | 19.6 | MUT |
| MDS | Th+An | *CBL* | 0.034 | 8.5 | 2.4 | MUT |
| MDS | Th+Ne | *SF3B1* | 0.007 | 0.0 | 34.4 | WT |

Ne: neutropenia; An: anemia; Th: thrombocytopenia; Pan: pancytopenia; Iso: isolated; MUT: mutated; WT: wildtype.

**Table S7. Risk scores of CCUS cases with available clinical follow-up data**

| Risk score | CCUS (n=73) |
| --- | --- |
| IPSS-R | **n=73** |
| Very low (VL) | 16 (22%) |
| Low (L) | 46 (63%) |
| Intermediate (I) | 7 (10%) |
| High (H) | 3 (4%) |
| Very high (VH) | 1 (1%) |
| IPSS-M | **n=73** |
| Very low (VL) | 17 (23%) |
| Low (L) | 41 (56%) |
| Moderate low (ML) | 5 (7%) |
| Moderate high (MH) | 6 (8%) |
| High (H) | 2 (3%) |
| Very high (VH) | 2 (3%) |
| CHRS | **n=65** |
| Low | 9 (14%) |
| Intermediate^pot*^ | 14 (21%) |
| Intermediate | 13 (20%) |
| High^pot*^ | 16 (25%) |
| High | 13 (20%) |

* CHRS could not be calculated more precisely due to missing values.

**Table S8. Risk scores of CCUS cases with available follow-up samples**

| Risk score | Progress (n=18) | Stable (n=21) |
| --- | --- | --- |
| IPSS-R | **n=18** | **n=21** |
| Very low (VL) | 0 (0%) | 3 (14%) |
| Low (L) | 13 (72%) | 16 (76%) |
| Intermediate (I) | 3 (17%) | 2 (10%) |
| High (H) | 2 (11%) | 0 (0%) |
| Very High (VH) | 0 (0%) | 0 (0%) |
| IPSS-M | **n=18** | **n=21** |
| Very low (VL) | 0 (0%) | 3 (14%) |
| Low (L) | 11 (61%) | 13 (62%) |
| Moderate low (ML) | 2 (11%) | 2 (9.5%) |
| Moderate high (MH) | 2 (11%) | 2 (9.5%) |
| High (H) | 1 (6%) | 1 (5%) |
| Very high (VH) | 2 (11%) | 0 (0%) |
| CHRS | **n=17** | **n=19** |
| Low | 1 (6%) | 4 (21%) |
| Intermediate^pot*^ | 1 (6%) | 3 (16%) |
| Intermediate | 0 (0%) | 5 (26%) |
| High^pot*^ | 7 (41%) | 4 (21%) |
| High | 8 (47%) | 3 (16%) |

* CHRS could not be calculated more precisely due to missing values.

**
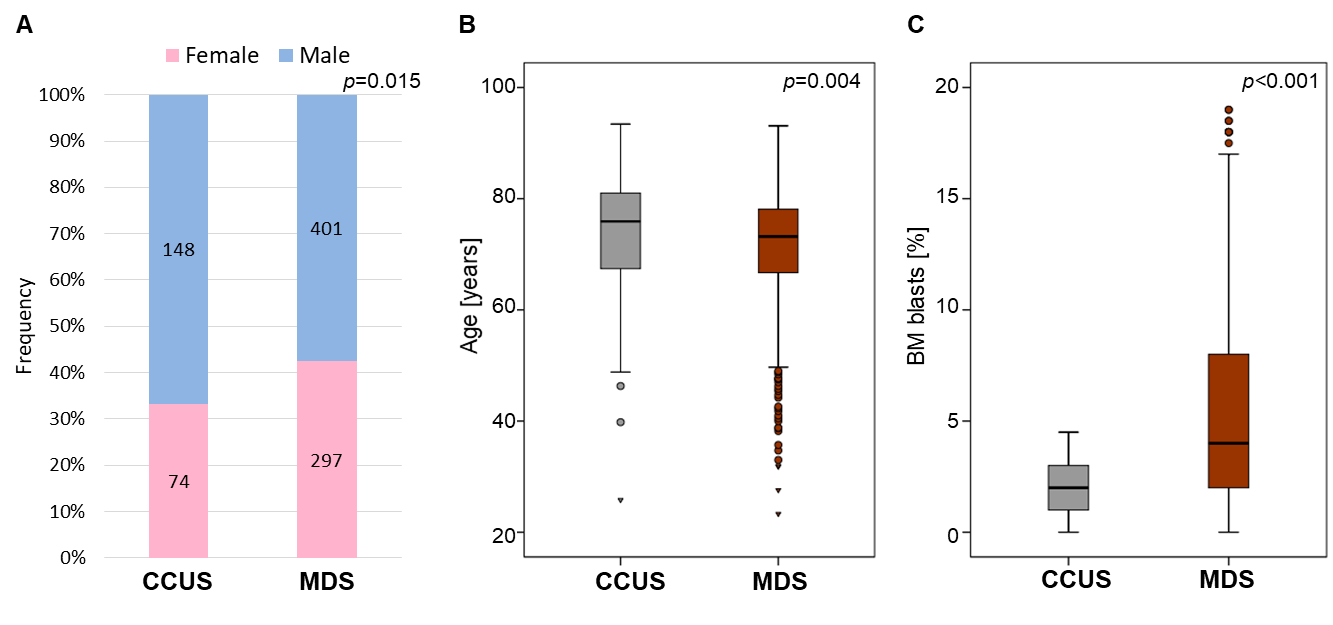
**

**Supplementary Figure S1: Baseline characteristics of CCUS and MDS.** Distribution of gender **(A)**, age **(B)** and BM blasts **(C)** within CCUS (n=222) and MDS (n=698). BM: bone marrow.

**
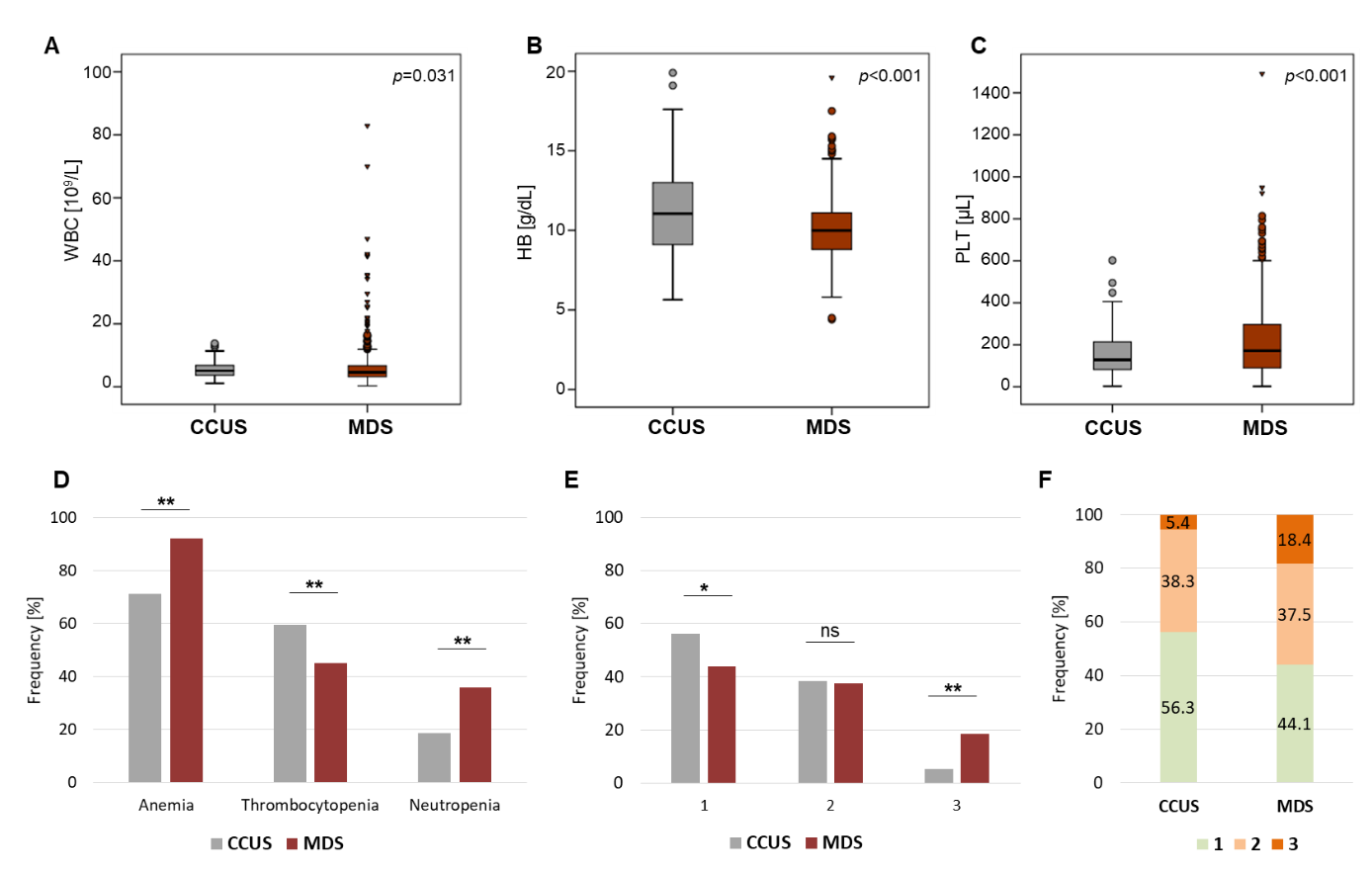
**

**Supplementary Figure S2: PB values within CCUS and MDS.** Distribution of WBC **(A)**, HB **(B)** and PLT **(C)** and detailed analysis of frequencies of types **(D)** and number **(E+F)** of cytopenias comparing CCUS (n=222) and MDS (n=651). PB: peripheral blood; WBC: white blood cells; HB: hemoglobin; PLT: platelets; * *p*<0.05; ***p*<0.001; ns: not significant.

**
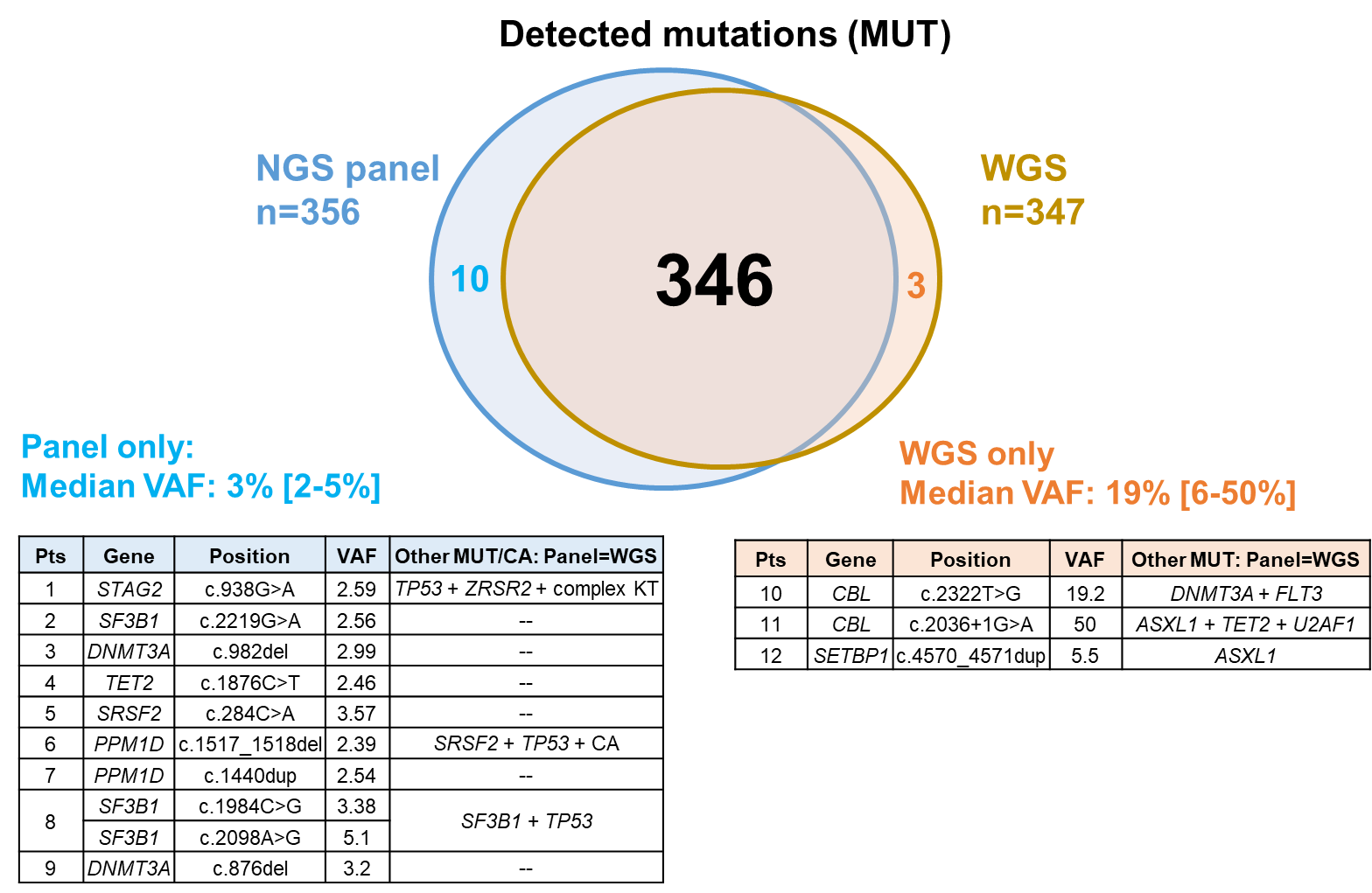
**

**Supplementary Figure S3: Mutational analysis of the CCUS cohort comparing NGS panel and WGS.** Overlap and discrepancies of MUT detection between sequencing techniques are shown (NGS panel only: n=10, blue; WGS only: n=3, orange)**.** MUT: mutation; VAF: variant allele frequency; CA: chromosomal abnormality; NGS: next generation sequencing; WGS: whole genome sequencing.

**
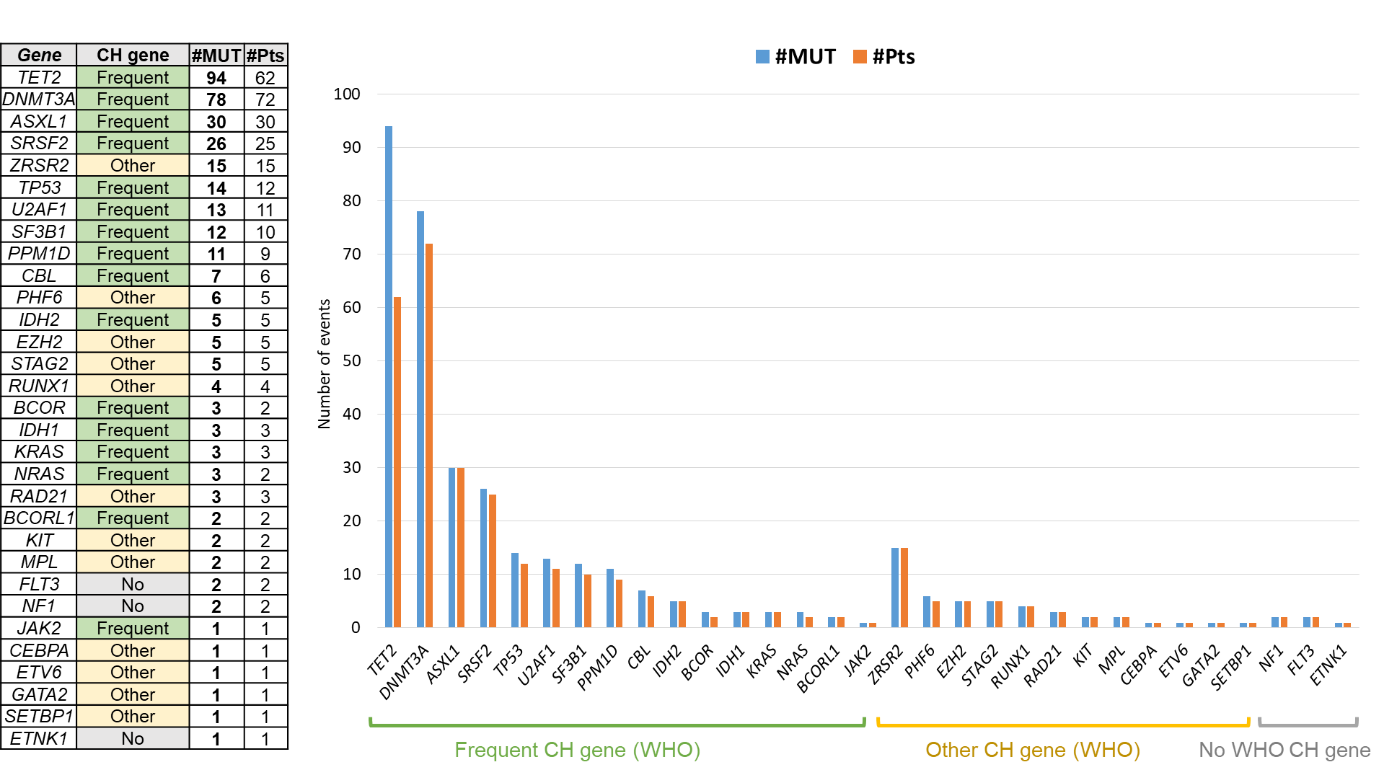
**

**Supplementary Figure S4: Analysis of detected mutations within the CCUS cohort (n=193 pts).** Total number mutations (blue bars) and number of patients with respective mutations (orange bars) are shown. Mutations were sorted by total number of mutation in the table and additionally by type of mutation in the graph. No WHO CH genes were required for IPSS-M calculation. CH: clonal hematopoiesis; MUT: mutation; Pts: patients.

**
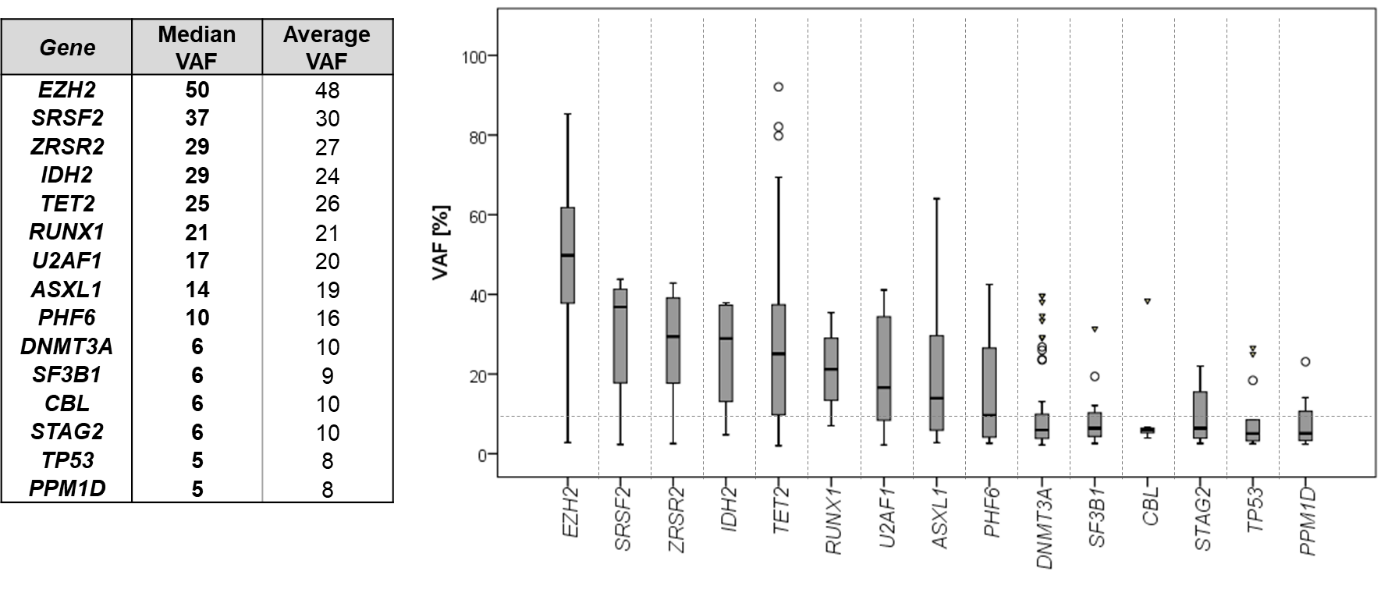
**

**Supplementary Figure S5: Top 15 mutated CCUS genes sorted by median VAF.** VAF: variant allele frequency; VAF of X-linked gene in males was corrected for gender; horizontal dashed line: 10% VAF.

**
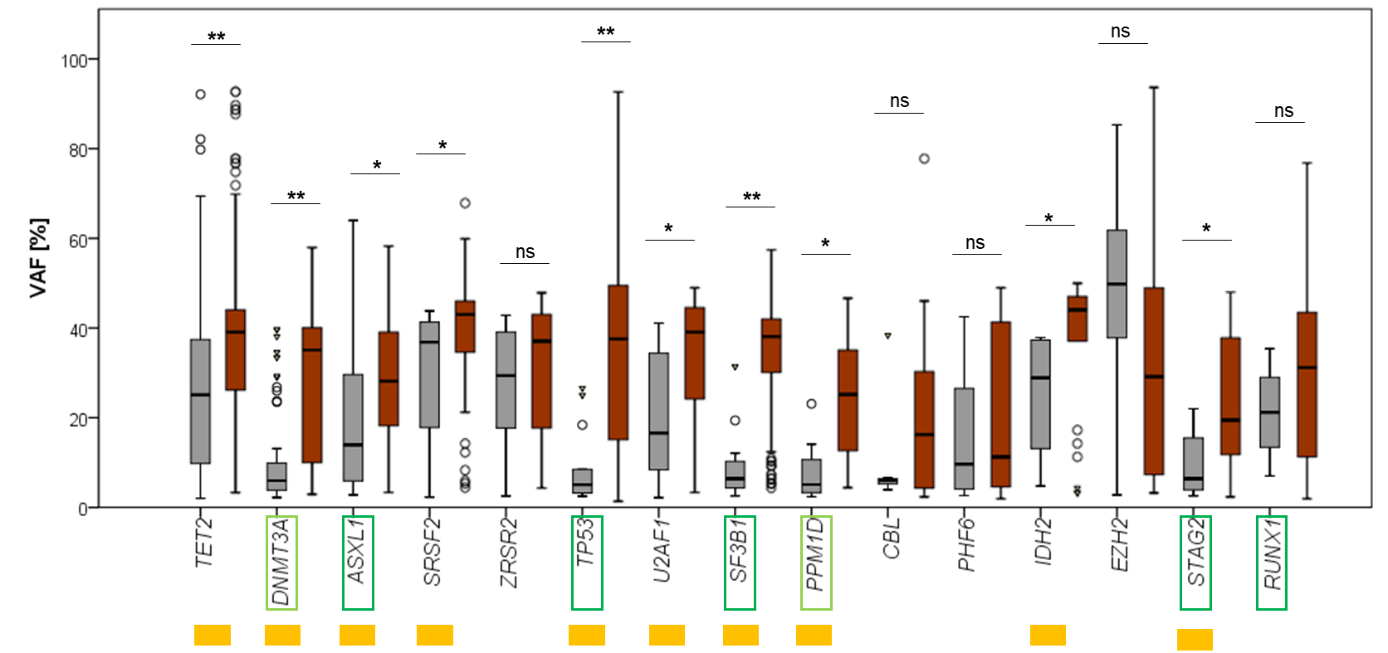
**

**Supplementary Figure S6: VAF of top 15 mutated CCUS genes within CCUS and MDS.** VAF of mutations were compared between CCUS (grey) and MDS (dark red). Mutations were sorted by CCUS mutational frequency. VAF: variant allele frequency; * *p*<0.05; ***p*<0.001; ns: not significant; yellow: significant higher VAF in MDS; VAF of X-linked genes in males was corrected for gender; light/dark green boxes: significantly more/less frequent in CCUS (see Figure 1).

**
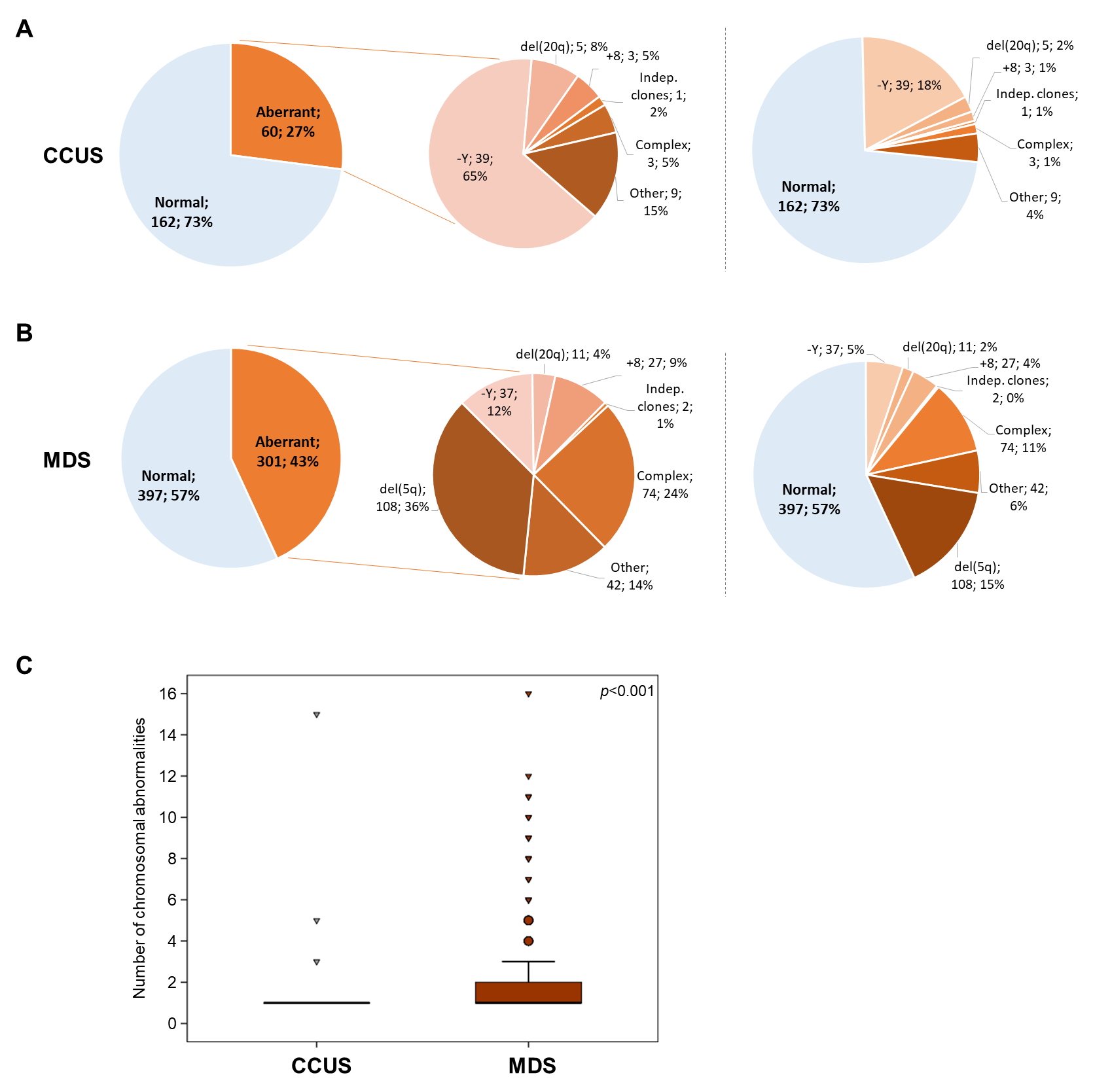
**

**Supplementary Figure S7**: **Cytogenetic analysis of CCUS and MDS.** Distribution of chromosomal abnormalities within CCUS **(A)** and MDS **(B).** Left charts: normal vs. aberrant karyotypes; middle charts: distribution of specific chromosomal abnormalities; right charts: combination of left and middle charts. Indep.: independent. **(C)** Boxplot showing the number of chromosomal abnormalities within CCUS and MDS cases.

**
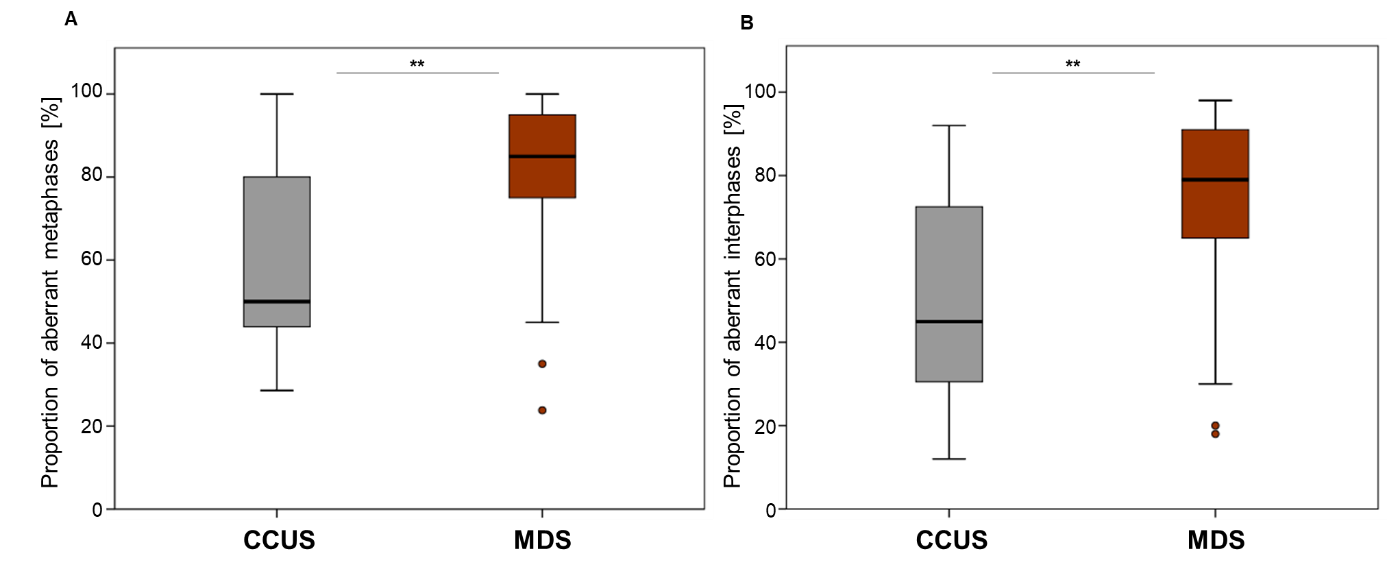
**

**Supplementary Figure S8. Cytogenetic analysis of cases with Y-loss.** CCUS (grey, n=39) and MDS (dark red: n=37) cases with Y-loss were analyzed by chromosome banding analysis **(A)** evaluating the proportion of aberrant metaphases and by FISH **(B)** evaluating the proportion of aberrant interphases. ***p*<0.001


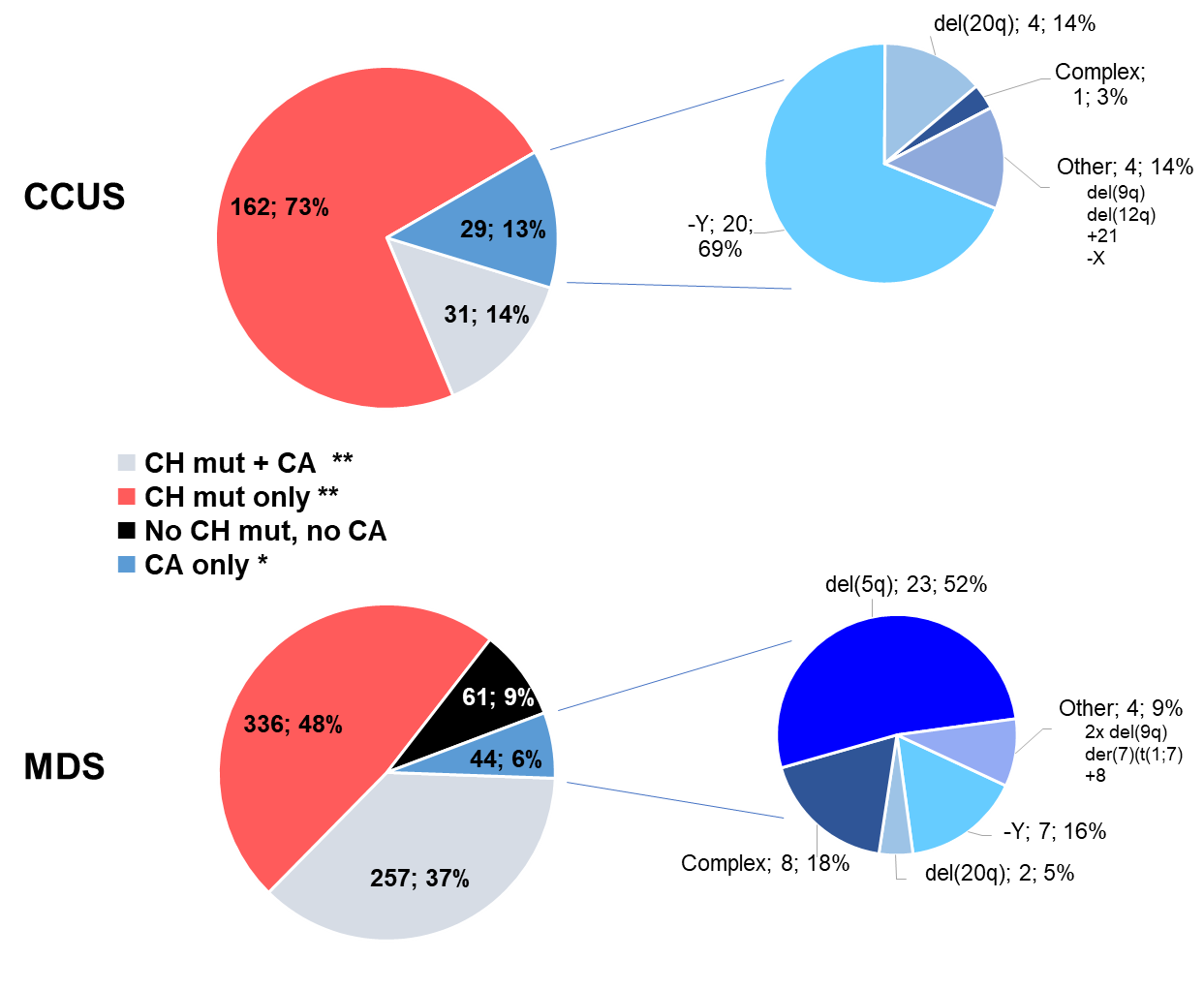


**Supplementary Figure S9. Analysis of clonality in CCUS and MDS.** Presence of chromosomal abnormalities (CA) and/or CH mutations within the CCUS (upper part) and MDS (lower part) cohort. Specific CA of cases without somatic mutations are shown in the right charts. CH: clonal hematopoiesis; MUT: mutation; ***p*<0.001; **p*<0.05

**
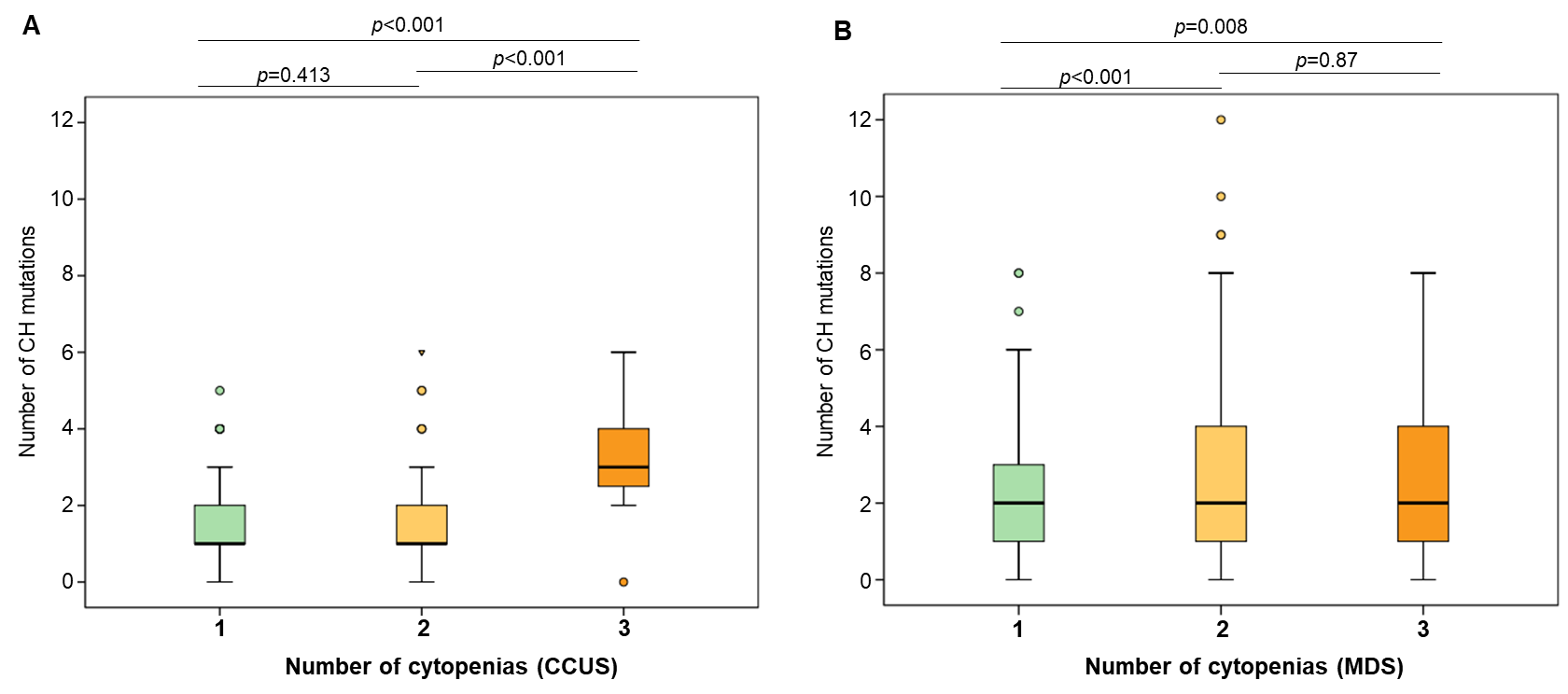
**

**Supplementary Figure S10: Relation between severity of cytopenia and number of CH mutations.** Boxplots showing the number of CH mutations for CCUS **(A)** and MDS **(B)** patients with respect to number of cytopenias. CH: clonal hematopoiesis.

**
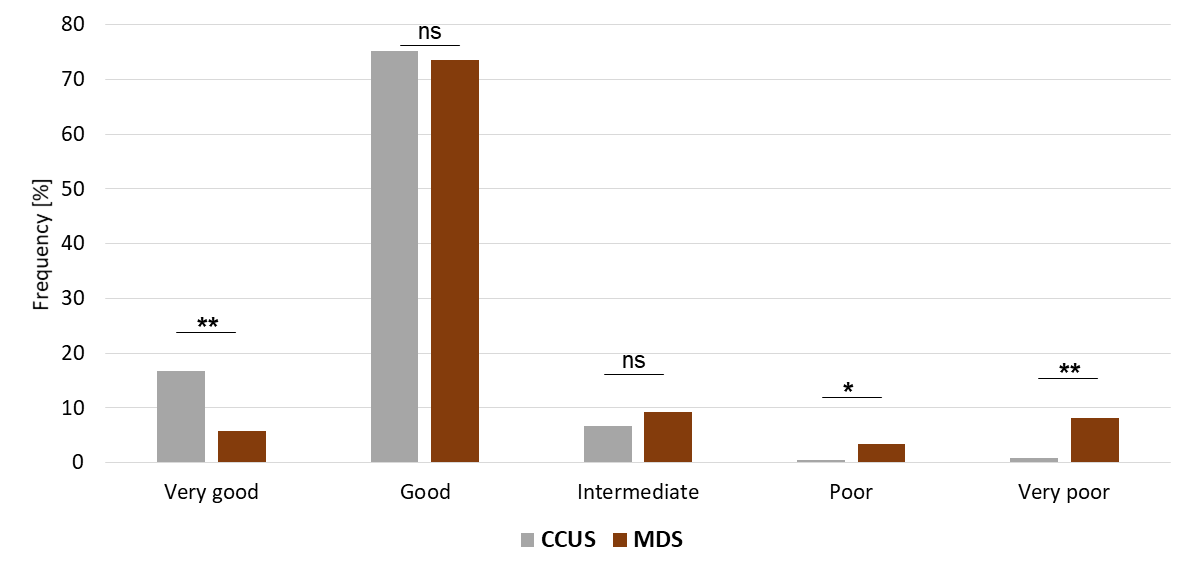
**

**Supplementary Figure S11: Frequency of IPSS-R cytogenetic risk groups.** The frequencies of the 5 IPSS-R cytogenetic risk groups are shown for CCUS (grey) and MDS (dark red) patients. ** *p*<0.001; * *p*<0.05; ns: not significant.

**
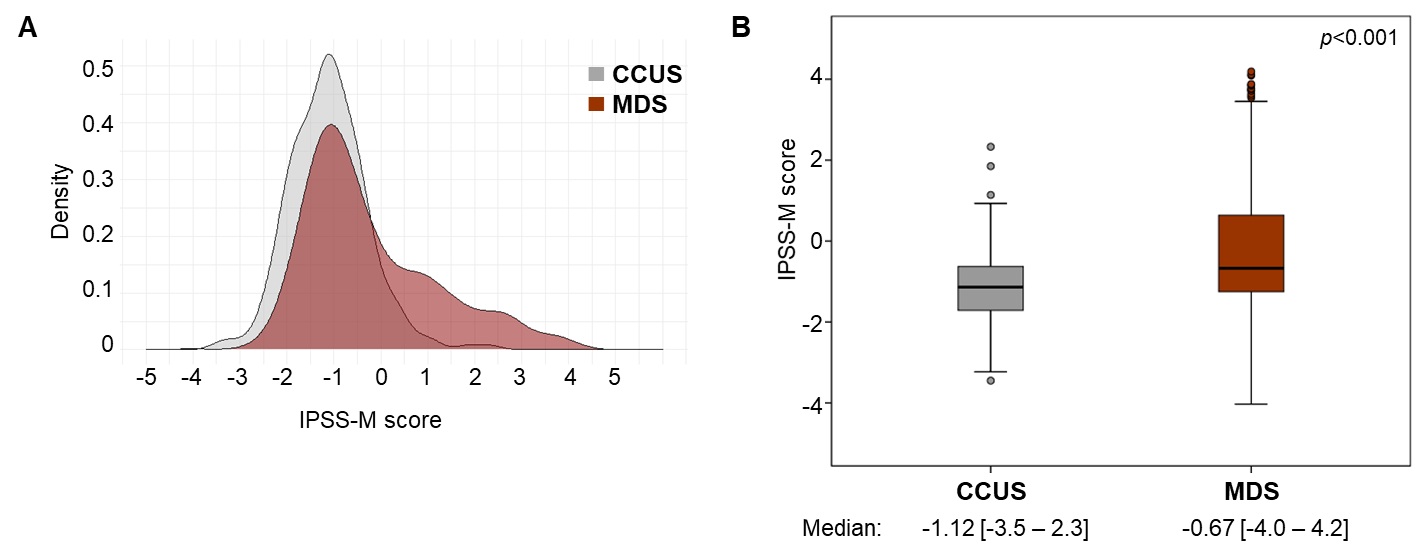
**

**Supplementary Figure S12: IPSS-M risk score of CCUS and MDS cases.** Densitiy plot **(A)** and box plot **(B)** showing the distribution of the IPSS-M scores within CCUS (grey) and MDS (dark red) patients.


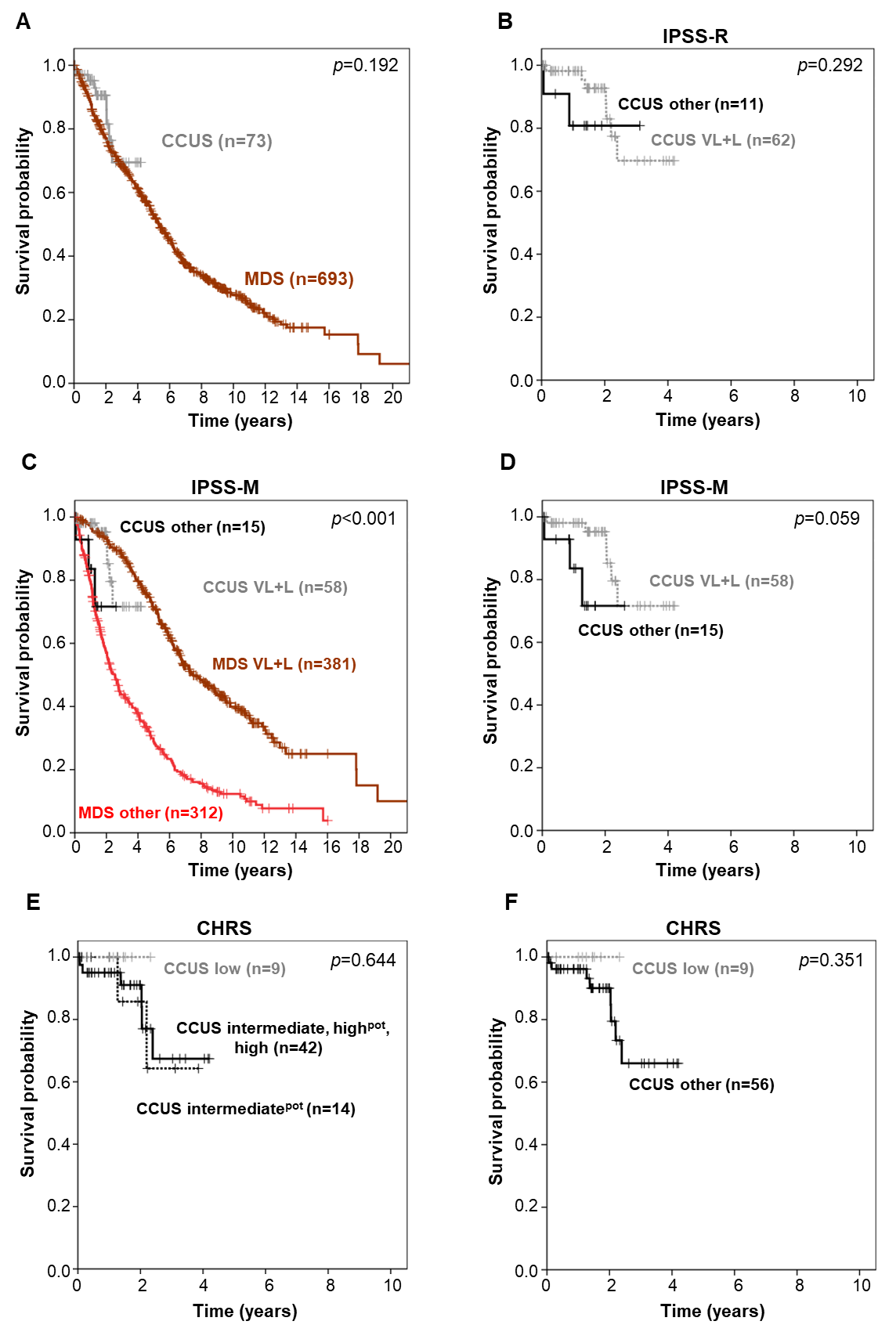


**Supplementary Figure S13: Overall survival analysis of CCUS and MDS cases. (A)** Overall survival (OS) analysis of CCUS compared to MDS (median OS: not reached vs. 5.3 years); median follow-up: 1.5 years (CCUS) and 9.3 (MDS) years. **(B)** OS analysis of CCUS cases grouped into very low (VL) or low (L) IPSS-R risk categories compared to others (intermediate, high, very high). **(C)** OS analysis of CCUS and MDS cases grouped into very low (VL) or low (L) IPSS-M risk categories compared to others (moderate low/high, high, very high). Median OS: 7.5 years for MDS VL+L and 2.5 years for MDS other. **(D)** OS analysis of CCUS cases grouped into very low (VL) or low (L) IPSS-M risk categories compared to others (moderate low/high, high, very high). **(E)** OS analysis of CCUS cases grouped into low or intermediate^pot^ CHRS risk categories compared to intermediate, high^pot^ and high. **(F)** OS analysis of CCUS cases grouped into low CHRS risk category compared to others (intermediate^pot^, intermediate, high^pot^, high).

**
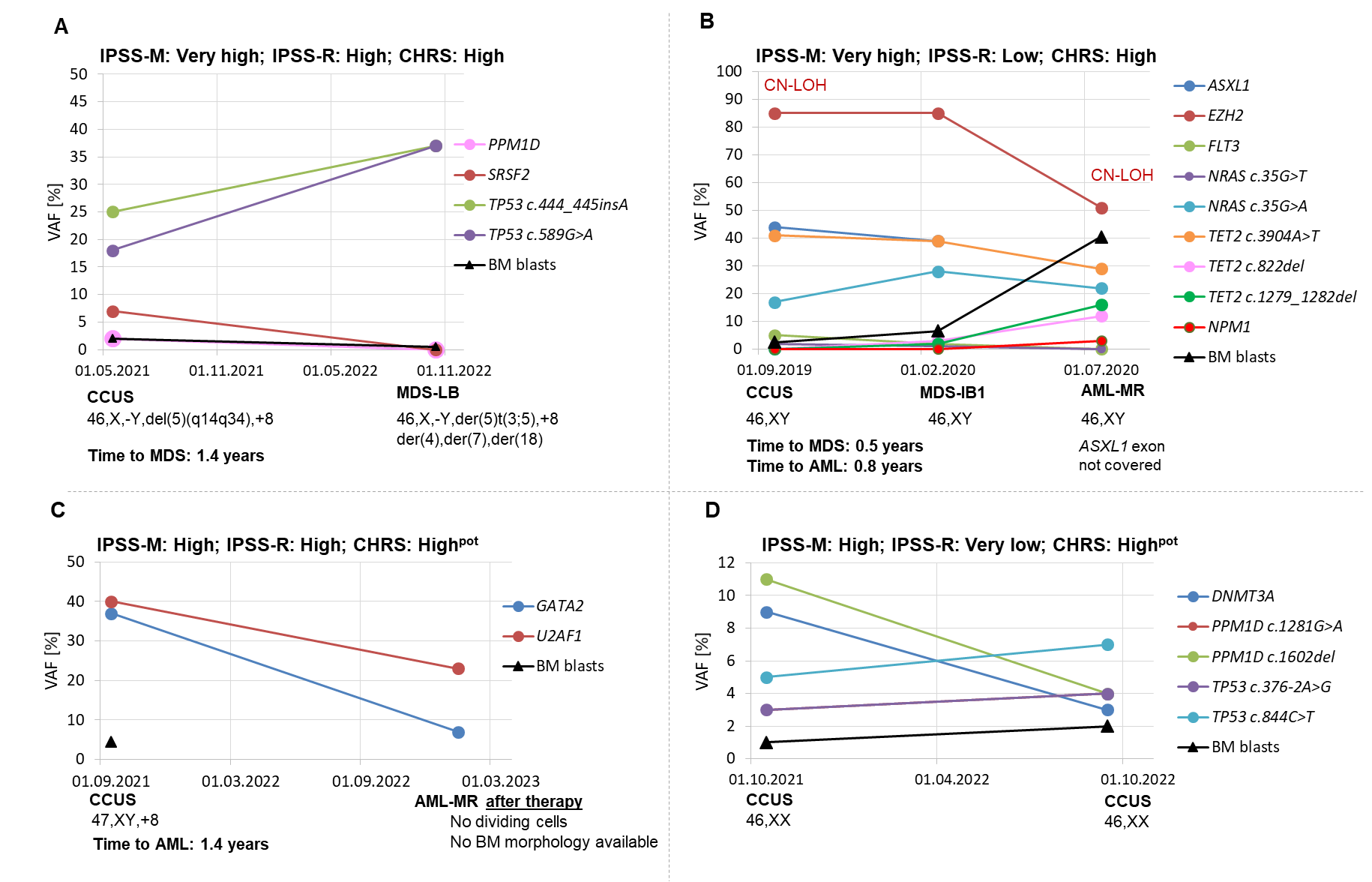
**

**Supplementary Figure S14: Disease courses of IPSS-M very high and high risk CCUS patients.** VAF of somatic mutations over time as well as cytogenetic information are shown for IPSS-M very high **(A+B)** and high **(C+D)** risk patients. VAF: variant allele frequency; CN-LOH: copy neutral loss of heterozygosity; LB: low blasts; IB: increased blasts; MR: myelodysplasia-related.

**
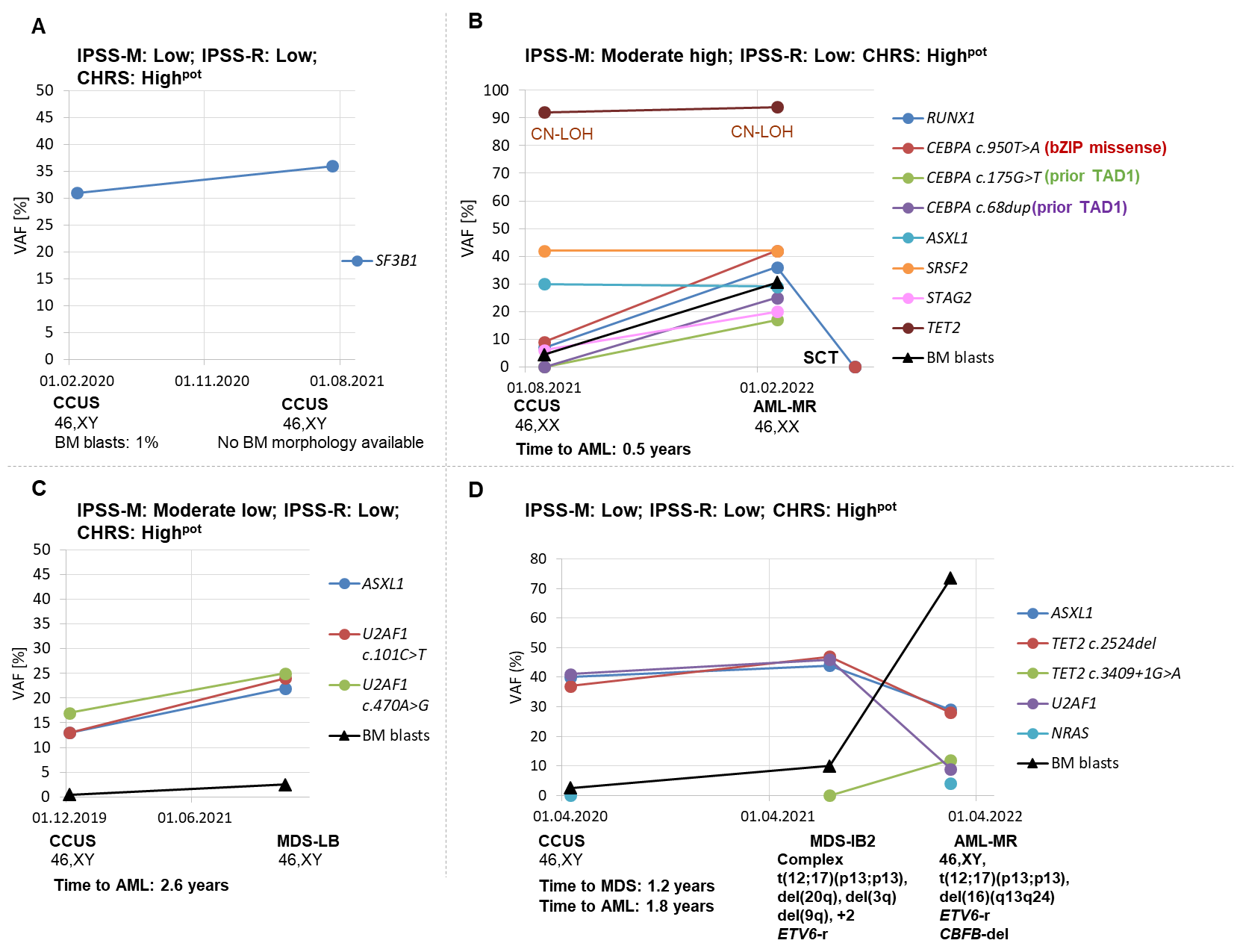
**

**Supplementary Figure S15. Disease courses of additional CCUS patients with molecular follow up data.** VAF of somatic mutations over time as well as cytogenetic information are shown for one patient remaining in stable disease **(A)** and three patients progressing to MDS and/or AML **(B-D)**. VAF: variant allele frequency; CN-LOH: copy neutral loss of heterozygosity; LB: low blasts; IB: increased blasts; MR: myelodysplasia-related; SCT: stem cell therapy; bZIP/TAD1: domains of *CEBPA*.

**References**

1. Huber S, Baer C, Hutter S, Dicker F, Meggendorfer M, Pohlkamp C, et al. AML classification in the year 2023: How to avoid a Babylonian confusion of languages. Leukemia. 2023;doi:10.1038/s41375-023-01909-w.

2. Haferlach T, Kern W, Schoch C, Hiddemann W, Sauerland MC. Morphologic dysplasia in acute myeloid leukemia: importance of granulocytic dysplasia. J Clin Oncol. 2003;21(15):3004-5.

3. Schoch C, Schnittger S, Bursch S, Gerstner D, Hochhaus A, Berger U, et al. Comparison of chromosome banding analysis, interphase- and hypermetaphase-FISH, qualitative and quantitative PCR for diagnosis and for follow-up in chronic myeloid leukemia: a study on 350 cases. Leukemia. 2002;16(1):53-9.

4. Maierhofer A, Mehta N, Chisholm RA, Hutter S, Baer C, Nadarajah N, et al. The clinical and genomic landscape of patients with DDX41 variants identified during diagnostic sequencing. Blood Adv. 2023; doi:10.1182/bloodadvances.2023011389.

5. Kern W, Voskova D, Schoch C, Hiddemann W, Schnittger S, Haferlach T. Determination of relapse risk based on assessment of minimal residual disease during complete remission by multiparameter flow cytometry in unselected patients with acute myeloid leukemia. Blood. 2004;104(10):3078-85.

6. Höllein A, Twardziok SO, Walter W, Hutter S, Baer C, Hernandez-Sanchez JM, et al. The combination of WGS and RNA-Seq is superior to conventional diagnostic tests in multiple myeloma: Ready for prime time? Cancer Genet. 2020;242:15-24.

7. Stengel A, Baer C, Walter W, Meggendorfer M, Kern W, Haferlach T, et al. Mutational patterns and their correlation to CHIP-related mutations and age in hematological malignancies. Blood Adv. 2021;5(21):4426-34.

8. Yang H, Chen G, Lima L, Fang H, Jimenez L, Li M, et al. HadoopCNV: A dynamic programming imputation algorithm to detect copy number variants from sequencing data. bioRxiv. 2017:124339.

9. Stengel A, Shahswar R, Haferlach T, Walter W, Hutter S, Meggendorfer M, et al. Whole transcriptome sequencing detects a large number of novel fusion transcripts in patients with AML and MDS. Blood Advances. 2020;4(21):5393-401.

10. Chen X, Schulz-Trieglaff O, Shaw R, Barnes B, Schlesinger F, Källberg M, et al. Manta: rapid detection of structural variants and indels for germline and cancer sequencing applications. Bioinformatics. 2016;32(8):1220-2.

11. Uhrig S, Ellermann J, Walther T, Burkhardt P, Fröhlich M, Hutter B, et al. Accurate and efficient detection of gene fusions from RNA sequencing data. Genome Research. 2021;31(3):448-60.

12. Haas BJ, Dobin A, Li B, Stransky N, Pochet N, Regev A. Accuracy assessment of fusion transcript detection via read-mapping and de novo fusion transcript assembly-based methods. Genome Biology. 2019;20(1):213.

13. Benjamini Y, Hochberg Y. Controlling the False Discovery Rate: A Practical and Powerful Approach to Multiple Testing. Journal of the Royal Statistical Society: Series B (Methodological). 1995;57(1):289-300.
